# The consistency of neuropathological diagnoses in patients undergoing surgery for suspected recurrence of glioblastoma

**DOI:** 10.1007/s11060-018-03037-3

**Published:** 2018-11-09

**Authors:** Matthias Holdhoff, Xiaobu Ye, Anna F. Piotrowski, Roy E. Strowd, Shannon Seopaul, Yao Lu, Norman J. Barker, Ananyaa Sivakumar, Fausto J. Rodriguez, Stuart A. Grossman, Peter C. Burger

**Affiliations:** 1 The Sidney Kimmel Comprehensive Cancer Center at Johns Hopkins, Johns Hopkins University School of Medicine, Baltimore, MD USA; 2 Department of Neurosurgery, Johns Hopkins University School of Medicine, Baltimore, MD USA; 3 Department of Neurology, Memorial Sloan Kettering Cancer Center, New York, NY USA; 4 Department of Neurology, Wake Forest School of Medicine, Winston-Salem, NC USA; 5 Department of Pathology, Johns Hopkins University School of Medicine, Baltimore, MD USA

**Keywords:** Pseudo-progression, Progression, Glioblastoma, Radiation necrosis, Treatment effect

## Abstract

**Purpose:**

Clinical factors and neuro-imaging in patients with glioblastoma who appear to progress following standard chemoradiation are unable to reliably distinguish tumor progression from pseudo-progression. As a result, surgery is commonly recommended to establish a final diagnosis. However, studies evaluating the pathologists’ agreement on pathologic diagnoses in this setting have not been previously evaluated.

**Methods:**

A hypothetical clinical history coupled with images of histological sections from 13 patients with glioblastoma who underwent diagnostic surgery for suspected early recurrence were sent to 101 pathologists from 50 NCI-designated Cancer Centers. Pathologists were asked to provide a final diagnosis (active tumor, treatment effect, or unable to classify) and to report on percent active tumor, treatment effect, and degree of cellularity and degree of mitotic activity.

**Results:**

Forty-eight pathologists (48%) from 30 centers responded. In three cases > 75% of pathologists diagnosed active tumor. In two cases > 75% diagnosed treatment effect. However, in the remaining eight cases the disparity in diagnoses was striking (maximum agreement on final diagnosis ranged from 36 to 68%). Overall, only marginal agreement was observed in the overall assessment of disease status [kappa score 0.228 (95% CI 0.22–0.24)].

**Conclusions:**

Confidence in any clinical diagnostic assay requires that very similar results are obtained from identical specimens evaluated by sophisticated clinicians and institutions. The findings of this study illustrate that the diagnostic agreement between different cases of repeat resection for suspected recurrent glioblastoma can be variable. This raises concerns as pathological diagnoses are critical in directing standard and experimental care in this setting.

**Electronic supplementary material:**

The online version of this article (10.1007/s11060-018-03037-3) contains supplementary material, which is available to authorized users.

## Introduction

Despite therapeutic advances over the past 2 decades, the prognosis of glioblastoma remains poor. Virtually all patients eventually progress after standard therapy with radiation and temozolomide, and succumb to their disease. Time of progression, however, is variable, and determination of disease status is required to select patients for second line therapies, including clinical trials. This is often challenging as treatment-related changes, commonly referred to as “pseudo-progression”, occur in 20–30% of patients and at a higher rate in patients with MGMT promoter methylation [1–5]. Unfortunately, contrast enhanced magnetic resonance imaging (MRI) as well as other advanced imaging techniques are currently unable to reliably differentiate true tumor progression from pseudo-progression [4–6]. As a result, the “gold standard” for assessment of disease status in many cases of presumed progression of glioblastoma rests on histopathological assessment. Several studies have suggested that pseudo-progression can be confirmed histologically, but determination of pseudo-progression versus active disease may or may not be prognostically significant [7–12].

Although considered the current “gold standard” for assessment of disease status, neuropathological assessment of recurrence can be difficult, as tissue frequently contains a mixture of treatment-related changes along with viable tumor in varying amounts [8, 10–12]. As a consequence, there can be discrepancies in the pathologic diagnoses when the same specimens are read by different neuropathologists. Incomplete tissue sampling can also be a confounding factor.

Confidence in a “standard” clinical diagnostic assay requires that similar results are obtained from identical specimens evaluated by different observers. As a result, we designed this survey to evaluate the consistency of a neuropathological diagnosis in patients who had completed standard radiation with concurrent temozolomide and subsequently underwent early surgery for a presumed recurrence of their glioblastoma.

## Methods

### Study objective

The primary objective of our study was to estimate the consistency of neuropathological diagnoses in patients undergoing surgery for presumed recurrence of glioblastoma.

### Study design

Survey material was sent by mail to 101 neuropathologists or pathologists who frequently read brain cancer cases at all National Cancer Institute (NCI)-designated cancer centers in the United States. The centers were contacted by the study team and names and contact information of the respective pathologists were collected. The material included: a hypothetical case history, the same for all cases, of a glioblastoma patient who underwent repeat neurosurgical resection within 1 year of diagnosis, a questionnaire (paper and pencil style, Table 1), as well as digital images of the neuropathology specimens from 13 patients who underwent repeat surgery for a presumed recurrence of their glioblastoma. Images were created from hematoxylin and eosin (H&E) stained formalin fixed paraffin embedded tissues. The number of images per case ranged from 4 to 8 (median 6). Images of special stains were not included.

**Table 1:** Case summary and questionnaire

Case summary
The patient is a 60 year-old who underwent gross total resection of a contrast enhancing right temporoparietal tumor, measuring 2.5 × 3.2 cm with surrounding edema and mass effect. Pathology was diagnostic for a glioblastoma (WHO grade IV) with brisk mitotic activity (Ki67 labeling index = 15%), necrosis, and microvascular proliferation. IDH1 was wild-type and *MGMT* promoter methylation was inconclusive. He underwent 6 weeks of standard radiation (60 Gy in 30 fractions) with daily concurrent temozolomide. He subsequently received four of the planned six cycles of standard adjuvant temozolomide (5 days/month at 150–200 mg/m^2^) with MRI scans every two months. After his fourth month of adjuvant temozolomide he complained of new headaches and left arm weakness that rapidly responded to moderate doses of dexamethasone. His MRI revealed a new focus of nodular contrast enhancement measuring 1.4 × 1.8 cm with surrounding T2/FLAIR changes and mass effect. The prior radiation fields were reviewed and it was determined that the entire area of new enhancement was treated with over 50 Gy. The patient was referred to surgery and underwent a second gross total resection. Tissue was sent to pathology. The neuro-oncology team is looking for guidance from the pathologist as to whether the new enhancing lesion is recurrent tumor, in which case the prior treatment would be deemed ineffective and novel therapies would be started, or whether this was pseudo-progression. The amount of active tumor present within the resected tissue is also of interest.
Questionnaire

Pathologists were asked to fill out one questionnaire for each of the 13 cases. They were asked to assign one of the following statements of overall disease activity to each of the cases: (1) “active tumor”, (2) “inactive tumor or treatment effect” or (3) “unable to classify”. In addition, they were asked to describe the “percent active tumor”, the “percent treatment effect”, the “cellularity” and the “mitotic activity” (Table 1). Finally, the participants were asked if they routinely read slides of patients with glioblastomas and if they were neuro-pathologists by training. Responses were returned anonymously to our study team by mail. This study was approved by the Institutional Review Board of the Johns Hopkins Medical Institutions.

### Selection and digital imaging of tissue slides

Thirteen patients with glioblastoma who had undergone early repeat neurosurgical resection for presumed recurrence were selected by P.C.B., neuropathologist on the study team. Cases were selected based on availability and presumed suitability for this survey, aiming at presenting a spectrum of disease activity and diagnostic difficulty that resembles a representative selection of cases in this clinical setting. The cases and microscopic fields thereof were selected to include a diversity of pathologic findings in the clinical setting of tumor recurrence. H&E stained histopathological slides were selected and photographed using a Zeiss Axio Imager 2.0, along with a Jenoptik ProgRes 14 digital camera (N.B., P.C.B., M.H.). Images were de-identified and stored in high-resolution (TIFF format) on USB drives.

### Statistical considerations

The survey contains five questions which are commonly considered by neuropathologists to determine tumor status during the disease process. Four questions were assigned with ordinal outcomes and one final question on pathology report sign-out was designed as a categorical outcome as active/or inactive/or unable to classify. The same five questions were applied to the 13 cases. All a questions are neither mutually exclusive nor complete overlapping. The summary results of the survey were presented at an individual case level due to highly varying interpretations of pathology reading among the 13 cases. The survey results were presented with standard descriptive summaries. Overall concordance among the pathologists on final pathology sign-out were assessed using Fleiss’s Kappa statistics [13]. Simpson’s Index was used to quantify the diversity of classification of the outcomes per each question among all the pathologists [14]. Pearson correlation coefficient was used to measure the relationship on individual question diversity to the final pathology sign-out. The survey was not powered with the intent to test hypotheses related to differences in pathological interpretations among the pathologists. As such, there is potential for extraneous differences between survey results, and all observed outcomes should be considered descriptive. All p-values were 2-sided.

## Results

### Survey participation

We received responses from 48 pathologists (48%), working at 30 centers. Forty-four (92%) of the respondents indicated that they were neuropathologists by training.

### Consistency in overall assessment of disease activity (question 5 of the questionnaire)

**Fig. 1:**
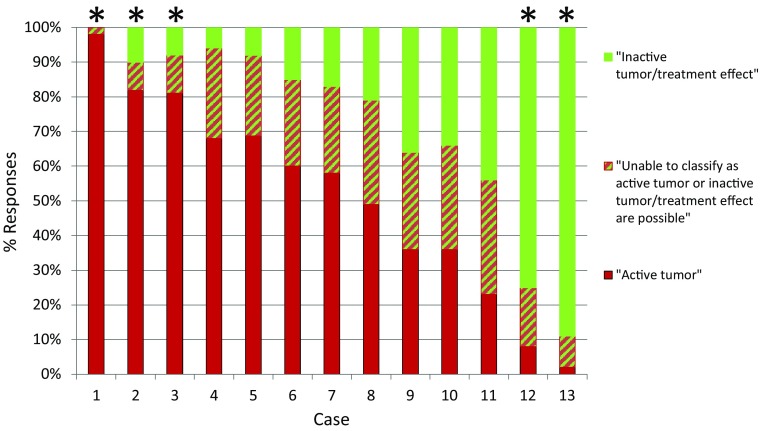
Overall assessment of disease activity as determined by participants in response to Question 5 of the survey. Cases are sorted from highest to lowest reported percentage of “active tumor”. Please note that cases were presented to survey participants in a different (random) order and not in the order presented in this figure. *Agreement on overall disease activity of > 75%

**Fig. 2:**
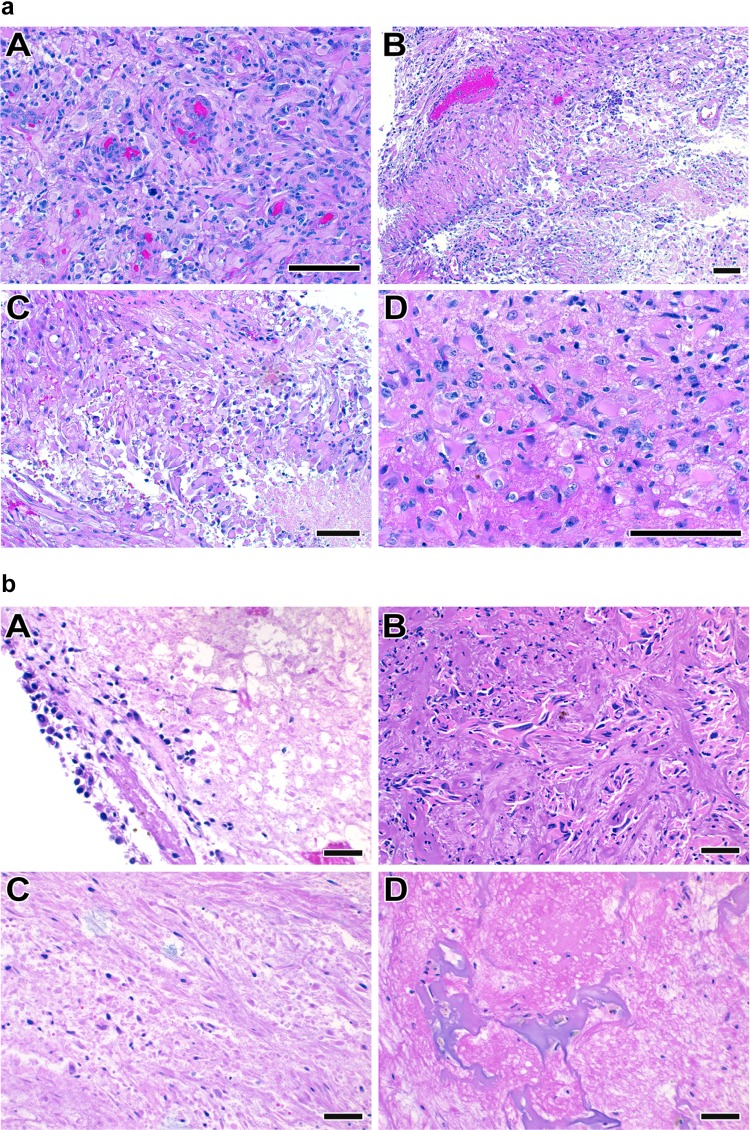
Histopathological images of a case with excellent agreement between reviewers. **a** strong agreement that this was active tumor (Case 1 in Fig. 1; scale bar represents 100 µm). **b** strong agreement that this was inactive tumor/treatment effect (Case 13 in Fig. 1; scale bare represents 50 µm)

**Fig. 3:**
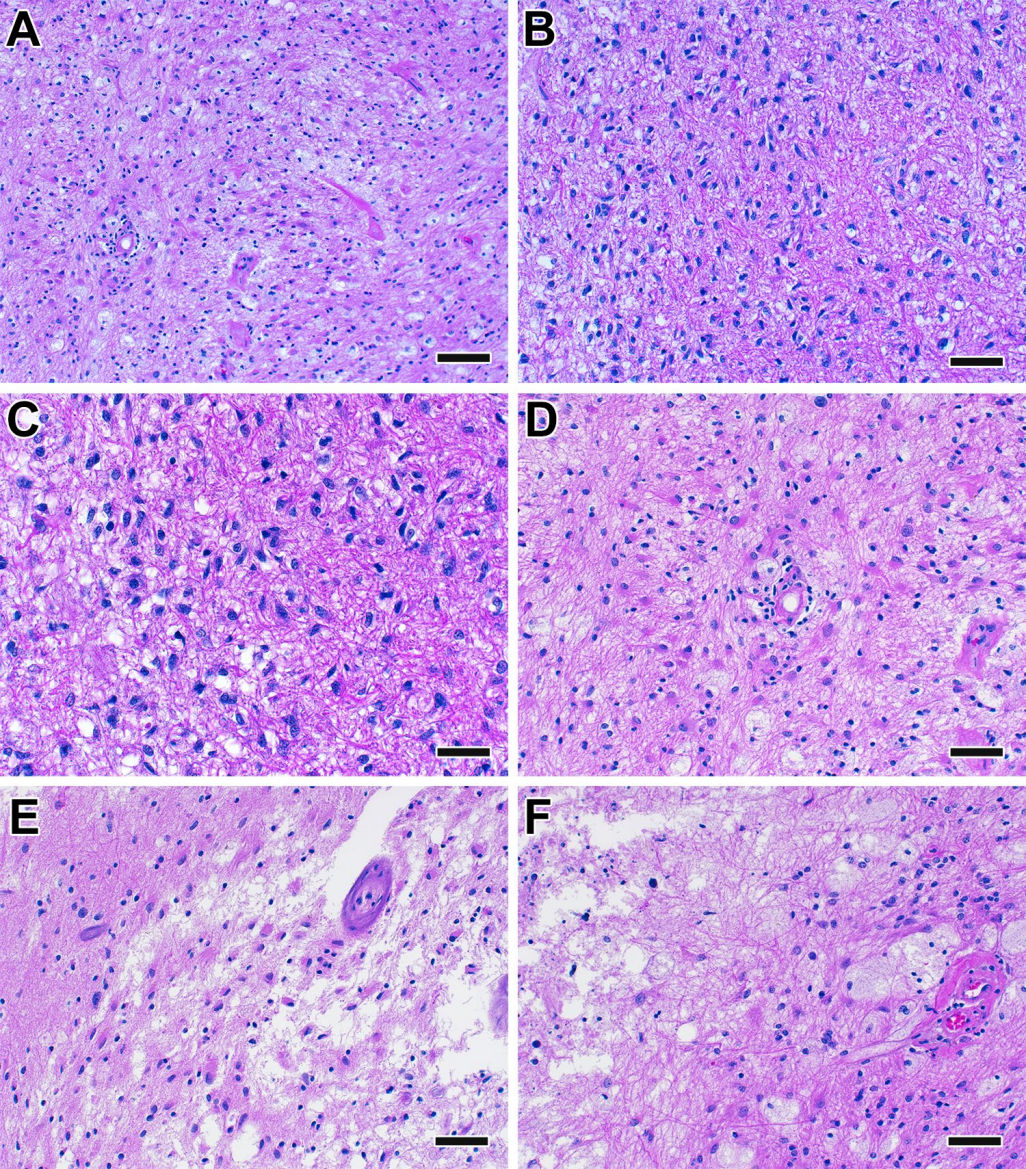
Histopathological images of a case with poor agreement (Case 9 in Fig. 1; scale bare represents 50 µm)

The primary objective of this study was to estimate the consistency of the final pathologic diagnoses in these 13 cases (Fig. 1). In five cases there was strong agreement on overall disease activity among participants, as defined as > 75% of the pathologists with the same diagnosis. Three of these five cases were determined to have “active disease” (cases 1–3) and two were diagnosed with “inactive disease/treatment effect” (cases 12, 13). The remaining eight of the 13 cases highlight differing degrees of agreement between pathologists and uncertainty regarding the underlying final diagnoses (cases 6–11). This is exemplified by case 9, in which 36% of the pathologists diagnosed the patient as having active tumor, 36% diagnosed the patient as having treatment effect and 28% were unable to make a diagnosis. The maximum agreement among all 13 cases ranged from 36 to 98% (median 68%). Representative examples are shown of cases with strong agreement between pathologists (Fig. 2) and one case of poor agreement (Fig. 3).

The agreement in overall assessment of disease activity within this survey, determined using Fleiss’ kappa statistics was 0.228 (95% CI 0.22–0.24). This is consistent only with “marginal agreement” between observers (kappa score interpretation: > 0.75 = excellent, 0.4 ≤ 0.75 = good, 0–0.4 = marginal reproducibility) [15].

### Different diagnostic parameters related to overall assessment of disease activity

**Fig. 4:**
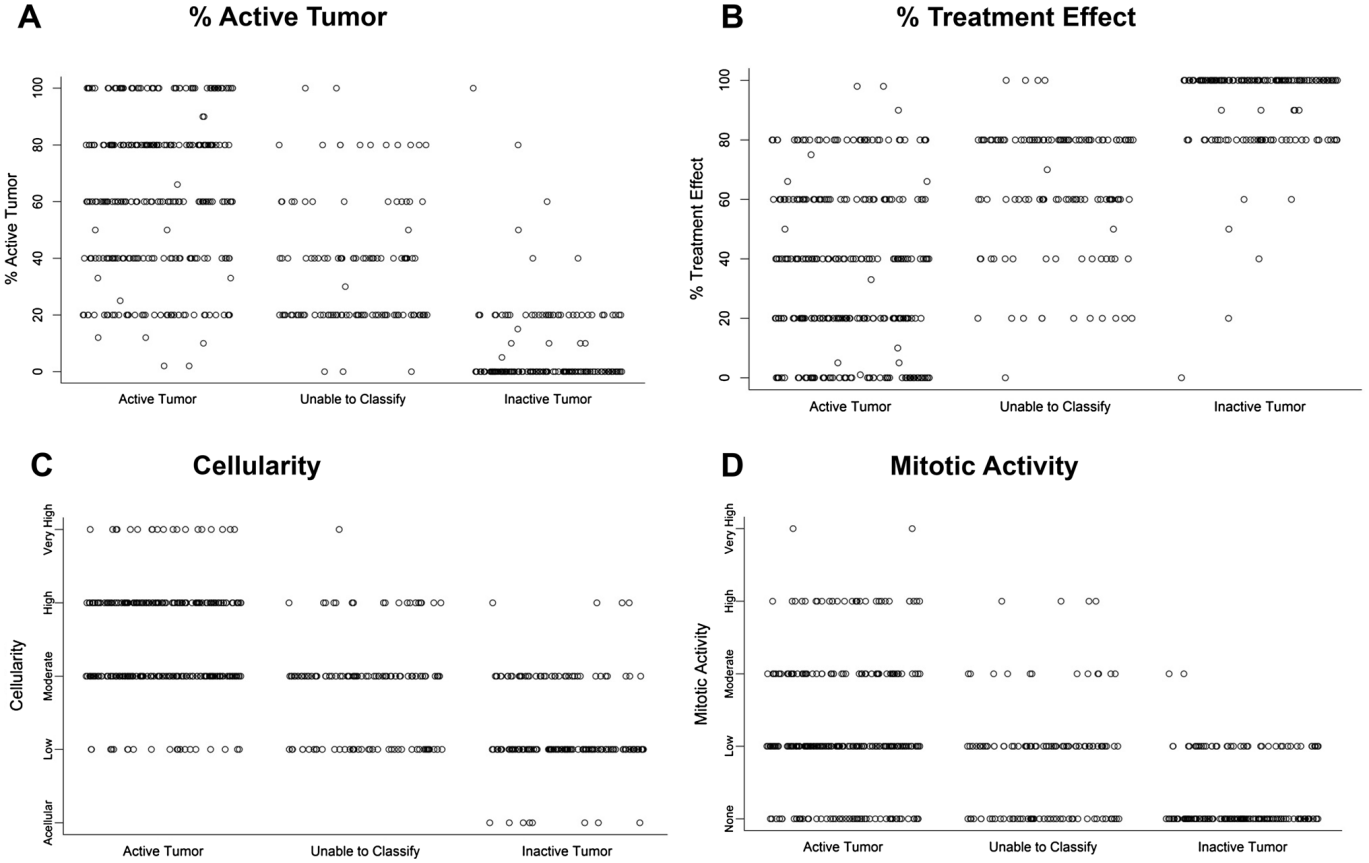
Four histological variabilities in relation to assigned category of disease activity. Each data point on the graph corresponds to one response to the survey. All responses in the survey were included. Y-axis: responses to survey questions about **a** percent active tumor, **b** percent treatment effect, **c** cellularity **d** mitotic activity. X-axis: Overall assessment of disease activity

To visualize the relationship between the different diagnostic parameters and the final assessment of disease status, all individual responses were plotted against overall assessment of disease status (Fig. 4a–d; for details of responses to different survey questions, please see Supplemental Data). Reported high percentages of “active tumor” per case were frequently associated with the overall assessment of disease status of “active disease” (Fig. 4a). Conversely, high percentages of “treatment effect” were often reported in association with “inactive tumor”. However, this figure also illustrates the diversity in responses between survey participants, including extreme outliers. Overall, it appears that a high percentage of treatment effect was needed for pathologists to report “inactive tumor” (Fig. 4b), whereas in some cases a relatively small percentage of active tumor was felt sufficient for the diagnosis of “active disease” (Fig. 4a). Responses regarding cellularity and mitotic activity follow a similar trend regarding their association with active disease versus treatment effect (Fig. 4b, c respectively).

## Discussion

Assessment of disease status in patients with presumed recurrence of glioblastoma remains a major challenge in neuro-oncology. As clinical histories, physical examinations, and advanced imaging studies fail to reliably distinguish active tumor from treatment effect, or pseudo-progression, patients require a surgical procedure to clarify the underlying cause of their deteriorating clinical and/or radiographic status. Prior studies have focused on the diagnostic challenges of histopathological determination of disease status at presumed recurrence, highlighting the importance of this issue. One of these studies demonstrated a relationship between diagnosis of disease activity and survival [8], when cases were distinguished into active tumor present in any amount versus no active tumor present. Other studies have failed to find consistent relationships between histopathological findings and outcome [9–12]. However inter-interpreter discrepancies in pathological interpretation have so far not been addressed.

This study had two principal findings. First, while some surgical specimens were generally agreed to have active tumor or no active tumor, there were other cases wherein the pathologic pictures were diverse. Highly variable diagnoses were the result. In the latter cases, there were almost equal distributions of responses between “active tumor”, “inactive tumor/treatment effect” and “unable to classify”. The second finding was the variable discordance between the percent of “active tumor” or “treatment effect” and the final pathologic diagnosis (Fig. 4).

Confidence in any “standard” clinical diagnostic assay requires that similar results are obtained from identical specimens evaluated by experienced interpreters. Certainly, diagnostic evaluation of tissue samples requires a degree of subjective distinction that may be difficult to standardize across diverse clinicians and institutions. However, the fact remains that there is a lack of diagnostic consistency in patients with glioblastoma who undergo surgery to establish a firm diagnosis after completing chemoradiation. The findings brought to light in this study have important implications, since an accurate pathologic diagnosis is critical in directing standard and experimental care in this setting. Ideally, a given test would reflect certainty about the underlying disease status with 100% sensitivity and specificity. However, as these criteria are difficult to meet in clinical practice, the best *available* test is often accepted as the “gold standard” [16]. The results of this survey suggest that uniform agreement is lacking among pathologists reading the same tissue sections. Entry into clinical trials would be directly affected if one pathologist designated a patient as having active tumor while another decided that the same specimen was not diagnostic for active tumor. Histopathology thus might not be predictable enough to be used as a final reference standard.

The problem of inter-interpreter consistency is not unique to neuro-oncology. One prominent example in which the lack of consistency in tissue testing provided a great challenge is Her-2 testing in patients with breast cancer. Testing and test interpretation was not uniform, and this problem was of great clinical relevance as presence or absence of Her-2 overexpression has direct implications for treatment and prognosis. Recognizing this problem, convened experts standardized the interpretation of Her-2, which in turn directly impacted clinical practice and research [17]. Similarly, there was uncertainty about the reproducibility of Gleason grading in prostate cancer, which is the basis for clinical staging and stratification of patients in clinical trials. Inter-observer studies eventually demonstrated that Gleason scoring was, for most prostate cancers, reproducible enough to use it as a reference standard [18].

The present study has several significant limitations. First, cases and images were selected based on availability and presumed suitability for this survey by one of the neuropathologists on the study team (PCB) and only a small number of cases (13) was included in this review. It needs to be stated that the selection of cases could certainly have influenced the study’s results and that it is possible that more complex cases were relatively over- or under-represented in this survey. Second, the way the survey was designed, did not truly represent a “real life” clinical scenario and it was somewhat artificial. For feasibility of this survey study, only digitalized images were sent to participants instead of actual glass slides and images selected for this study represented only a small cross-sectional area of the entire specimen. Moreover, immunohistochemical stains and proliferation markers such as Ki67 or the mitosis marker p-HH3 were not included in the survey material. Third, differential understanding of the terminology could have affected inter-observer variability in responses. This is in part as “active tumor”, “inactive tumor” and “treatment effect” are not well established in the literature and as the participants had not been provided with a clear definition as part of this survey. The reason for only providing these three answers was to make survey participants “commit” to a summary diagnosis of overall disease activity or to state that they were unable to classify the case. This was felt to be of importance as differentiation of active versus inactive disease is critically relevant for therapeutic decisions. The survey findings illustrate that such a dichotomization of results was not feasible in all cases that were part of this survey. In contrast to this survey, in current clinical practice, neuropathologists have a variety of ways to formulate a clinical impression of presence, absence and relative abundance of tumor, inactive tumor and treatment effect. Fourth, there may be considerable variability in interpretation of pathology slides between neuropathologists versus general pathologists and regarding variable levels of experience. This has not been captured in our study as we sent the survey only to pathologists that were routinely interpreting glioblastoma specimens at the respective NCI-designated cancer centers, 92% of whom were neuropathologists by training. The issue of different level of specialization and experience between pathologists will need to be carefully considered in future studies addressing this clinical topic. In addition, future studies looking at this clinical question should include higher numbers of cases than in this pilot survey study and cases should be selected in an unbiased way, for example by selection of consecutive cases as they present in clinical practice and ideally between several participating institutions.

We do not believe that these limitations negate the message that histopathology, at least as applied to tumors sampled by the surgeons in this study, may not be consistent enough to be a reliable reference standard. Across multiple institutions, formal criteria need to be developed to assure more uniform and reproducible diagnosis in patients who are undergoing repeat surgery for presumed progression of disease. Given the complexity and importance of this topic, we feel that this challenge be best taken on by an expert committee such as the recently launched subcommittee by the Response Assessment in Neuro-Oncology (RANO) group, in order to assure input from neuropathologists from different institutions and other clinical disciplines involved in the treatment of malignant gliomas. Aspects to consider when developing these criteria will include optimization of tissue sampling (sending the complete tissue to pathology), as well as new or improved strategies for quantification of viable tumor within a given sample, including immunohistochemistry, next generation sequencing and proteomics. Once a new set of criteria are proposed, these should be rigorously tested, initially using a training set and then, for validation, in an adequately powered prospective clinical study.

## Electronic supplementary material

Below is the link to the electronic supplementary material.

Supplementary material 1 (DOCX 34 KB)
