# Supplementary material for: The consistency of neuropathological diagnoses in patients undergoing surgery for suspected recurrence of glioblastoma
Source: J Neurooncol. 2018 Nov 9;141(2):347–54. doi: 10.1007/s11060-018-03037-3 (PMC6342857; doi:10.1007/s11060-018-03037-3)
Supplement: Supplementary file 1 — Supplementary material 1 (DOCX 34 KB) [file 11060_2018_3037_MOESM1_ESM.docx]

Supplemental Data

Holdhoff et al.: The consistency of neuropathological diagnoses in patients undergoing surgery for suspected recurrence of glioblastoma

**I. Responses to individual survey questions:**

***Survey Question 1: What percent of the available tissue contained active tumor?***

Frequency N (%)

| Case # | 0% | 20% | 40% | 60% | 80% | 100% | Total  responses |
| --- | --- | --- | --- | --- | --- | --- | --- |
| 1 | 0 (0) | 1 (2.22) | 2 (4.44) | 3 (6.67) | 14 (31.11) | 25 (55.56) | 45 (100) |
| 2 | 4 (8.51) | 4 (8.51) | 0 (0) | 4 (8.51) | 9 (19.15) | 26 (55.32) | 47 (100) |
| 3 | 1 (2.22) | 14 (31.11) | 9 (20) | 13 (28.89) | 8 (17.78) | 0 (0) | 45 (100) |
| 4 | 3 (6.25) | 22 (45.83) | 8 (16.67) | 9 (18.75) | 6 (12.5) | 0 (0) | 48 (100) |
| 5 | 3 (6.67) | 4 (8.89) | 12 (26.67) | 10 (22.22) | 14 (31.11) | 2 (4.44) | 45 (100) |
| 6 | 15 (34.88) | 17 (39.53) | 3 (6.98) | 5 (11.63) | 3 (6.98) | 0 (0) | 43 (100) |
| 7 | 3 (6.52) | 8 (17.39) | 23 (50) | 10 (21.74) | 2 (4.35) | 0 (0) | 46 (100) |
| 8 | 4 (8.7) | 0 (0) | 1 (2.17) | 4 (8.7) | 28 (60.87) | 9 (19.57) | 46 (100) |
| 9 | 34 (79.07) | 6 (13.95) | 0 (0) | 0 (0) | 2 (4.65) | 1 (2.33) | 43 (100) |
| 10 | 2 (4.55) | 10 (22.73) | 11 (25) | 17 (38.64) | 4 (9.09) | 0 (0) | 44 (100) |
| 11 | 13 (28.89) | 9 (20) | 12 (26.67) | 2 (4.44) | 8 (17.78) | 1 (2.22) | 45 (100) |
| 12 | 25 (59.52) | 13 (30.95) | 3 (7.14) | 1 (2.38) | 0 (0) | 0 (0) | 42 (100) |
| 13 | 7 (16.28) | 27 (62.79) | 7 (16.28) | 2 (4.65) | 0 (0) | 0 (0) | 43 (100) |

***Survey Question 2: What percent of the available tissue contained inactive tumor/treatment effect?***

Frequency N (%)

| Case # | 0% | 20% | 40% | 60% | 80% | 100% | Total  responses |
| --- | --- | --- | --- | --- | --- | --- | --- |
| 1 | 31 (70.45) | 8 (18.18) | 2 (4.55) | 2 (4.55) | 1 (2.27) | 0 (0) | 44 (100) |
| 2 | 29 (61.7) | 9 (19.15) | 0 (0) | 0 (0) | 4 (8.51) | 5 (10.64) | 47 (100) |
| 3 | 0 (0) | 9 (19.57) | 14 (30.43) | 8 (17.39) | 14 (30.43) | 1 (2.17) | 46 (100) |
| 4 | 0 (0) | 7 (14.58) | 8 (16.67) | 8 (16.67) | 23 (47.92) | 2 (4.17) | 48 (100) |
| 5 | 2 (4.35) | 14 (30.43) | 11 (23.91) | 12 (26.09) | 3 (6.52) | 4 (8.7) | 46 (100) |
| 6 | 0 (0) | 3 (6.67) | 7 (15.56) | 2 (4.44) | 18 (40) | 15 (33.33) | 45 (100) |
| 7 | 0 (0) | 2 (4.35) | 12 (26.09) | 22 (47.83) | 7 (15.22) | 3 (6.52) | 46 (100) |
| 8 | 9 (20) | 28 (62.22) | 3 (6.67) | 1 (2.22) | 0 (0) | 4 (8.89) | 45 (100) |
| 9 | 1 (2.17) | 1 (2.17) | 0 (0) | 0 (0) | 8 (17.39) | 36 (78.26) | 46 (100) |
| 10 | 0 (0) | 4 (8.89) | 18 (40) | 11 (24.44) | 10 (22.22) | 2 (4.44) | 45 (100) |
| 11 | 1 (2.22) | 8 (17.78) | 3 (6.67) | 11 (24.44) | 10 (22.22) | 12 (26.67) | 45 (100) |
| 12 | 0 (0) | 0 (0) | 1 (2.17) | 4 (8.7) | 12 (26.09) | 29 (63.04) | 46 (100) |
| 13 | 0 (0) | 0 (0) | 3 (6.52) | 8 (17.39) | 27 (58.7) | 8 (17.39) | 46 (100) |

***Survey Question 3: How would you describe the cellularity in this specimen?***

Frequency N (%)

| **Case #** | **“acellular”** | **“low”** | **“moderate”** | **“high”** | **“very high”** | **Total** |
| --- | --- | --- | --- | --- | --- | --- |
|  |  |  |  |  |  | **responses** |
| **1** | 0 (0) | 0 (0) | 4 (8.51) | 33 (70.21) | 10 (21.28) | 47 (100) |
| **2** | 0 (0) | 6 (12.5) | 29 (60.42) | 11 (22.92) | 2 (4.17) | 48 (100) |
| **3** | 0 (0) | 8 (17.39) | 29 (63.04) | 9 (19.57) | 0 (0) | 46 (100) |
| **4** | 0 (0) | 12 (25) | 30 (62.5) | 6 (12.5) | 0 (0) | 48 (100) |
| **5** | 0 (0) | 4 (8.33) | 36 (75) | 8 (16.67) | 0 (0) | 48 (100) |
| **6** | 0 (0) | 27 (56.25) | 21 (43.75) | 0 (0) | 0 (0) | 48 (100) |
| **7** | 0 (0) | 3 (6.82) | 6 (13.64) | 30 (68.18) | 5 (11.36) | 44 (100) |
| **8** | 0 (0) | 0 (0) | 7 (15.22) | 35 (76.09) | 4 (8.7) | 46 (100) |
| **9** | 6 (13.33) | 37 (82.22) | 2 (4.44) | 0 (0) | 0 (0) | 45 (100) |
| **10** | 0 (0) | 5 (11.11) | 26 (57.78) | 14 (31.11) | 0 (0) | 45 (100) |
| **11** | 0 (0) | 19 (41.3) | 27 (58.7) | 0 (0) | 0 (0) | 46 (100) |
| **12** | 2 (4.26) | 43 (91.49) | 2 (4.26) | 0 (0) | 0 (0) | 47 (100) |
| **13** | 0 (0) | 14 (30.43) | 29 (63.04) | 3 (6.52) | 0 (0) | 46 (100) |

***Survey Question 4: How would you describe the mitotic activity in this specimen?***

Frequency N (%)

| Case # | “none” | “low” | “moderate” | “high” | “very high” | Total responses |
| --- | --- | --- | --- | --- | --- | --- |
| 1 | 0 (0) | 9 (19.15) | 18 (38.3) | 18 (38.3) | 2 (4.26) | 47 (100) |
| 2 | 14 (29.79) | 26 (55.32) | 5 (10.64) | 2 (4.26) | 0 (0) | 47 (100) |
| 3 | 17 (38.64) | 23 (52.27) | 3 (6.82) | 1 (2.23) | 0 (0) | 44 (100) |
| 4 | 14 (31.11) | 28 (62.22) | 3 (6.67) | 0 (0) | 0 (0) | 45 (100) |
| 5 | 11 (24.44) | 29 (64.44) | 5 (11.11) | 0 (0) | 0 (0) | 45 (100) |
| 6 | 23 (48.94) | 21 (44.68) | 3 (6.38) | 0 (0) | 0 (0) | 47 (100) |
| 7 | 8 (19.51) | 18 (43.9) | 10 (24.39) | 5 (12.2) | 0 (0) | 41 (100) |
| 8 | 3 (6.98) | 17 (39.53) | 16 (37.21) | 7 (16.28) | 0 (0) | 43 (100) |
| 9 | 40 (86.96) | 6 (13.04) | 0 (0) | 0 (0) | 0 (0) | 46 (100) |
| 10 | 15 (34.09) | 22 (50) | 6 (13.64) | 1 (2.27) | 0 (0) | 44 (100) |
| 11 | 24 (55.81) | 18 (41.86) | 1 (2.33) | 0 (0) | 0 (0) | 43 (100) |
| 12 | 38 (80.85) | 9 (19.15) | 0 (0) | 0 (0) | 0 (0) | 47 (100) |
| 13 | 24 (53.33) | 17 (37.78) | 3 (6.67) | 1 (2.22) | 0 (0) | 45 (100) |

***Survey Question 5: How would you sign out this pathology?***

Frequency N (%)

| Case # | “Active tumor” | “Inactive tumor/treatment effect” | “Unable to classify as active tumor or inactive tumor/treatment effect is possible” | Total responses |
| --- | --- | --- | --- | --- |
| 1 | 46 (97.87) | 0 (0) | 1 (2.13) | 47 (100) |
| 2 | 39 (81.25) | 5 (10.42) | 4 (8.33) | 48 (100) |
| 3 | 29 (60.42) | 7 (14.58) | 12 (25) | 48 (100) |
| 4 | 23 (48.94) | 10 (21.28) | 14 (29.79) | 47 (100) |
| 5 | 28 (58.33) | 8 (16.67) | 12 (25) | 48 (100) |
| 6 | 11 (22.92) | 21 (43.75) | 16 (33.33) | 48 (100) |
| 7 | 32 (68.09) | 4 (8.51) | 11 (23.4) | 47 (100) |
| 8 | 38 (80.85) | 4 (8.51) | 5 (10.64) | 47 (100) |
| 9 | 1 (2.13) | 42 (89.36) | 4 (8.51) | 47 (100) |
| 10 | 32 (68.09) | 3 (6.38) | 12 (25.53) | 47 (100) |
| 11 | 17 (36.17) | 16 (34.04) | 14 (29.79) | 47 (100) |
| 12 | 4 (8.33) | 36 (75) | 8 (16.67) | 48 (100) |
| 13 | 17 (36.17) | 17 (36.17) | 13 (27.66) | 47 (100) |

**II. Supplemental Biostatistical Data:**

**Fleiss’ Kappa Statistics:**

Fleiss’ Kappa was used to assess an overall agreement on pathology sign-out of tumor classification among the all pathologists. There is a marginal agreement on the tumor classification among the pathologists, Kappa 0.228 (95%CI: 0.22-0.24).

**Reference:** Fleiss, J. L. (1971) "Measuring nominal scale agreement among many raters." Psychological Bulletin, Vol. 76, No. 5 pp. 378–382.
